# Age at developmental cortical injury differentially Alters corpus callosum volume in the rat

**DOI:** 10.1186/1471-2202-8-94

**Published:** 2007-11-12

**Authors:** Steven W Threlkeld, Glenn D Rosen, R Holly Fitch

**Affiliations:** 1 Department of Psychology; Behavioral Neuroscience Division, University of Connecticut, 806 Babbidge Road, Storrs, CT 06269-4154, USA; 2 Department of Neurology, Beth Israel Deaconess Medical Center, 330 Brookline Ave, Boston, MA 02215, USA; 3 Harvard Medical School, Boston, MA 02115, USA

## Abstract

**Background:**

Freezing lesions to developing rat cortex induced between postnatal day (P) one and three (P1 – 3) lead to malformations similar to human microgyria, and further correspond to reductions in brain weight and cortical volume. In contrast, comparable lesions on P5 do not produce microgyric malformations, nor the changes in brain weight seen with microgyria. However, injury occurring at all three ages does lead to rapid auditory processing deficits as measured in the juvenile period. Interestingly, these deficits persist into adulthood only in the P1 lesion case [1]. Given prior evidence that early focal cortical lesions induce abnormalities in cortical morphology and connectivity [1-4], we hypothesized that the differential behavioral effects of focal cortical lesions on P1, P3 or P5 may be associated with underlying neuroanatomical changes that are sensitive to timing of injury. Clinical studies indicate that humans with perinatal brain injury often show regional reductions in corpus callosum size and abnormal symmetry, which frequently correspond to learning impairments [5-7]. Therefore, in the current study the brains of P1, 3 or 5 lesion rats, previously evaluated for brain weight, and cortical volume changes and auditory processing impairments (P21-90), were further analyzed for changes in corpus callosum volume.

**Results:**

Results showed a significant main effect of Treatment on corpus callosum volume [F (1,57) = 10.2, P < .01], with lesion subjects showing significantly smaller callosal volumes as compared to shams. An Age at Treatment × Treatment interaction [F(2,57) = 3.2, P < .05], indicated that corpus callosum size decreased as the age of injury decreased from P5 to P1. Simple effects analysis showed significant differences between P1 and P3 [F(1,28) = 8.7, P < .01], and P1 and P5 [F(1,28) = 15.1, P < .001], subjects. Rats with P1 injury resulting in microgyria had the greatest reduction in corpus callosum volume (22% reduction), followed by the P3 group (11% reduction), which showed a significant reduction in corpus callosum volume compared to shams [F(1,31) = 5.9, P < .05]. Finally, the P5 lesion group did not significantly differ from the sham subjects in callosal volume.

**Conclusion:**

Decrements in corpus callosum volume in the P1 and 3 lesion groups are consistent with the reductions in brain weight and cortical volume previously reported for microgyric rats [1,8]. Current results suggest that disruption to the cortical plate during early postnatal development may lead to more widely dispersed neurovolumetric anomalies and subsequent behavioral impairments [1], compared with injury that occurs later in development. Further, these results suggest that in a human clinical setting decreased corpus callosum volume may represent an additional marker for long-term behavioral outcome.

## Background

Freezing lesions to the developing cortical plate result in a cascade of local and distal anatomical and physiological changes, including hyperexcitability around the point of disruption, deviation of axonal projections from targets in the hemisphere contralateral to the pathology, and changes in thalamo-cortical connectivity [2-4]. Freezing lesions induced on postnatal day 1 (P1) and P3 in rats resemble human four-layer microgyria (as seen postmortem in dyslexics; [9,10]). Moreover, the presence of microgyria has been associated with rapid auditory processing impairments in rats [1,11,12]. Given evidence that disruptions in auditory processing may contribute to disruptions in language development [13,14], human microgyria could relate to human dyslexia, at least in part, through similar auditory processing disruptions [1,11,12].

Previous research indicates that injury to developing cortex during peak periods of neuronal migration results in greater decreases in brain weight and cortical volume as compared to injury occurring beyond the cessation of neuronal migration in rats (around P4; [1]). In fact, focal freezing lesions on P5 do not lead to significant decreases in brain weight, or cortical volume, nor the formation of four-layer microgyria as seen in P1 and 3 lesion cases. Surprisingly, focal injury on P1, 3 or 5 does lead to deficits in processing brief gaps in white noise as measured during the juvenile period (P23-39), regardless of the presence/absence of microgyria or changes in cortical volume/brain weight observed. However, when rapid auditory processing was assessed in adult rats (P60-64), only subjects with lesions induced on P1 (who had cortical microgyria) were found to exhibit persistent rapid auditory processing deficits [1]. These data suggested that despite early behavioral impairments seen with focal lesions on P1-5, cortical disruption specifically during neuronal migration appears to exert more pronounced and long-term behavioral and neuroanatomical effects as compared to injury occurring after the completion of neuronal migration.

In terms of human research, neuromorphometric studies investigating the effects of developmental pathology on long-term behavioral outcome are scarce. However, recent studies suggest a link between early cortical developmental malformations and learning-related cognitive impairments (i.e., reading and language impairments; [15,9]. Moreover, human data also suggests that changes in key markers of developmental disruption such as abnormal hemispheric asymmetry and reduced brain, corpus callosum, and cortical volumes, may provide insight into the location and timing of pathological interference that leads to the behavioral expression of learning impairments such as dyslexia [5,6,15-17].

Changes in anatomical structures such as the corpus callosum may provide a marker for the timing of pathological onset and/or the extent of developmental disruption. As such, the current study sought to determine the effects of developmentally induced bilateral focal freezing lesions (P1, 3, 5 and sham) on corpus callosum volume in rats previously assessed for auditory processing, brain weight and cortical volume [1]. We predicted that changes in corpus callosum volume would correspond with the profile of change previously seen for brain weight and cortical volume [1], and that these changes would in turn reflect long-term behavioral outcome.

## Results

**Figure 1:**
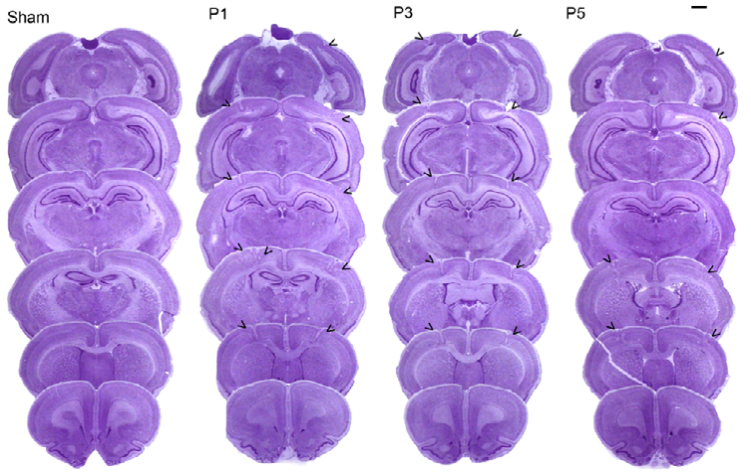
Photomicrographs (1.3×) showing six typical coronal sections, for each age of injury condition (P1, 3 and 5) and sham brains, used for point counting. Arrowheads indicate extensive microgyria in the P1 and 3 cases and subtle laminar disruption in the P5 lesion condition. Scale bar is 1 mm.

Post mortem histological analysis (P90+ tissue) revealed no evidence of cortical damage in any of the sham subjects (n = 16). Post mortem analysis showed the presence of double bilateral microgyria only in the P1 (n = 13) and P3 (n = 17) lesion groups. The P5 (n = 17) lesion group, which received comparable freezing lesion treatments relative to the P1 and P3 groups, did not show evidence of microgyria. However, the P5 lesion group did show some disrupted cortical lamination in areas of cortex directly underlying the probe application points. Lesions were seen mostly in sensorimotor cortex (SM-I), with some extension into frontal, temporal, or occipital cortices. The majority of double lesions in P1 and P3 conditions appeared as one continuous severe microgyric lesion. However, the P5 group showed a pattern of disruption that was centered on the specific areas of probe application. This pattern appeared as four relatively small distinct pockets of displaced cortical lamination resulting from the freezing insults (Figure 1).

### Corpus Callosum volume

**Figure 2:**
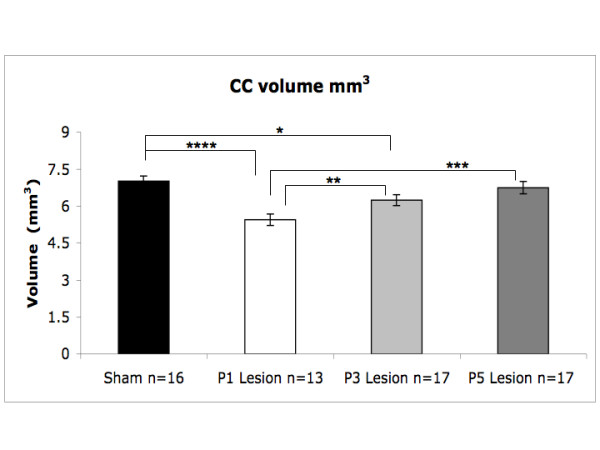
Histograms showing corpus callosum volumes (mm^3^) for P1, 3, 5 lesion, and sham subjects. Stars indicate level of significance (* = .05, ** = .01, *** = .001, **** = .0001).

A univariate ANOVA was computed for corpus callosum volume, using Age at Treatment (3 levels; P1, 3, 5) and Treatment (2 levels; Sham, Lesion) as fixed factors (Figure 2). Results showed a significant main effect of Treatment on corpus callosum volume [F (1,57) = 10.2, P < .01], with lesion subjects showing significantly smaller callosal volumes as compared to shams. An Age at Treatment × Treatment interaction was also found for corpus callosum volume [F(2,57) = 3.2, P < .05], indicating that corpus callosum volumes were smaller as the age of injury decreased from P5 to P1. Among lesion subjects, simple effects analyses revealed significant differences between P1 and P3 [F(1,28) = 8.7, P < .01], and P1 and P5 [F(1,28) = 15.1, P < .001], subjects. There was no significant difference between P3 and P5 lesion subjects [F(1,32) = 2.3, P = .13 (ns)]. However, unlike the P5 lesion group [F(1,31) = .53, P = ns], P3 subjects did show a significant reduction in corpus callosum volume compared to shams (11% reduction relative to shams) [F(1,31) = 5.9, P < .05], indicating an overall reduction in corpus callosum volume similar to, but not as great as, the reduction seen in the P1 lesion condition (22% reduction relative to shams) [F(1,27) = 24.8, P < .0001].

## Discussion

The current findings show that focal bilateral freezing lesions to the developing cortex result in differential reductions in corpus callosum volume as a function of the timing of the insult. Results show an age of treatment by treatment interaction, indicating that as the age at injury moves from P5 to P1 the corpus callosum becomes smaller. This progressive decrease in corpus callosum volume is evidenced by smaller callosal volumes in P1 versus P3 lesion, and P3 lesion versus sham subjects. Although the cause of these changes is unknown, the current results add to previous studies showing reduced cortico-cortical and thalamo-cortical connectivity, along with reductions in cortical volume and brain weight, resulting from P1 lesion-induced microgyria [1,4,18]. As previously reported for the subjects in the current analysis, brain weight and cortical volume decrease as a function of the age at which injury occurs. Specifically, P1 induced lesion subjects had the smallest cortex and brain weight compared to shams, followed by P3, and then the P5 lesion group (which did not differ from shams; [1]). All of these data taken together suggest that changes in corpus callosum volume, in addition to cortical volume and brain weight, may represent an important clinical marker for the timing of cortical developmental pathology, which may contribute to some aspects of learning impairment. Moreover, the current report provides increased support for the hypothesis that early injury to developing cortex can have marked effects on the volume of various structures directly and indirectly related to the point of disruption.

We previously reported that P1 focal injury to cortex led to long-term impairment in processing short but not long duration gaps in white noise using a startle response paradigm [1]. Further, prior to the current analysis all subjects received a total of 10 days of silent gap/reflex modification testing [1]. During the juvenile period (P23-39), all subjects in the current brain assessment received 8 days of silent gap testing, in addition to 2 days of silent gap testing in adulthood (P60-64). Briefly, acoustic testing involved the placement of rats on a load cell platform while a pseudo randomized set of variable duration silent gaps was presented in continuous 75 dB broadband white noise prior to a 105 dB startle eliciting noise burst. Detection of the silent gap cue elicited a reduction in the startle response relative to an uncued trial, where no gap preceded the startle burst. Importantly, subjects with the largest reduction in corpus callosum volume (P1 lesion group), as seen in the current analysis, showed the worst long-term behavioral outcome [1]. Specifically, P1 lesion subjects continued to show impairments in 2–10 ms silent gap detection after P60, whereas P3 and P5 lesion groups no longer showed the robust pattern of impairment (for further detail see [1]). It is important to note that all of the subjects evaluated in the current study received the same testing experience prior to sacrifice. Further, the nature of reflex modification insured that alternative strategies could not be used as with more complex maze learning or operant conditioning tasks. While the possibility exists that behavioral testing effected the brains of the three lesion groups or shams differently, the fact that P5 and sham subjects did not differ in corpus callosum volume suggests that age at injury was an important factor in determining long-term neuromorphological profiles across groups. Further, even with repeated behavioral testing these results are especially important within the clinical context. However, future studies will seek to determine the effects of different types of experience on key anatomical markers, such as corpus callosum volume.

Although the results of the current study do not address the causal mechanisms underlying auditory processing impairments, they may represent an additional marker for the presence of developmental pathology which might be involved in the appearance of human learning impairments such as dyslexia, [13,14,19,20], as well as cortical developmental malformations [9,15,16]. In recent years, as neuroimaging has become more accessible to researchers, the corpus callosum has gained increased attention as a target for possible pathology underlying developmental learning impairments [5-7]. However, sampling in these studies is frequently limited to young adults and there is often little information regarding the timing or occurrence of pre/pari natal insults. Therefore, the use of rodent models of focal cortical injury such as the one presented here may help identify possible windows during brain development at which particular structures are more susceptible to degradation, which in turn could lead to more pronounced long term behavioral pathology. The present data supports the notion that assessment of colossal morphology in populations at risk for neurodevelopmental pathologies (e.g., vascular hemorrhage, focal ischemia), may help identify whether and when injury occurred, as well as predict potential long-term behavioral outcomes.

## Conclusion

The reductions in corpus callosum volume in the P1 and 3 lesion groups are consistent with the reductions in brain weight and cortical volume previously reported for microgyric rats [1,8]. Current results suggest that disruption to the cortical plate during early postnatal development may lead to more widely dispersed neurovolumetric anomalies and subsequent behavioral impairments [1], compared with injury that occurs later in development. Further, these results suggest that in a human clinical setting decreased corpus callosum volume may represent an additional marker for long-term behavioral outcome.

## Methods

### Subjects

Subjects included 63 male Wistar rats born to purchased dams (Charles River Laboratory, Wilmington, MA) at the University of Connecticut. All procedures were conducted in compliance with the National Institutes of Health Guide for the Care and Use of Laboratory Animals, including adequate measures to minimize pain and discomfort. The Institutional Animal Care and Use committee (IACUC) at the University of Connecticut approved all procedures.

### Induction of freezing lesion

On the day of surgery litters were culled to 10 pups (8 male, 2 female), with male pups randomly assigned to receive double-pair freezing lesion or sham surgery. Females were retained to equalize litter size and avoid all-male litters. Surgeries involved the placement of a 2 mm stainless steel probe cooled to -70°C on the skull-cap. Focal lesions were induced as a double-pair (two to each hemisphere) as previously described [11]. This procedure was modeled after that developed by Dvorak and colleagues [10,21,22]. P1 rats received the freezing probe for 5 seconds, while the P3 animals were given 7 seconds and the P5 rats a 10 second lesion (to compensate for age-related increases in thickness of the skull). A subset of sham subjects was taken from each age group (P1, P3, P5), and received similar treatment with a room temperature probe. After surgery, pups were individually marked with ink footpad injections, warmed under a lamp and placed back with the mother. Final n's for each group were: sham P1 (n = 5), sham P3 (n = 6), sham P5 (n = 5), (total sham= 16); P1 lesion (n = 13); P3 lesion (n = 17); P5 lesion (n = 17).

### Brain analysis

**Figure 3:**
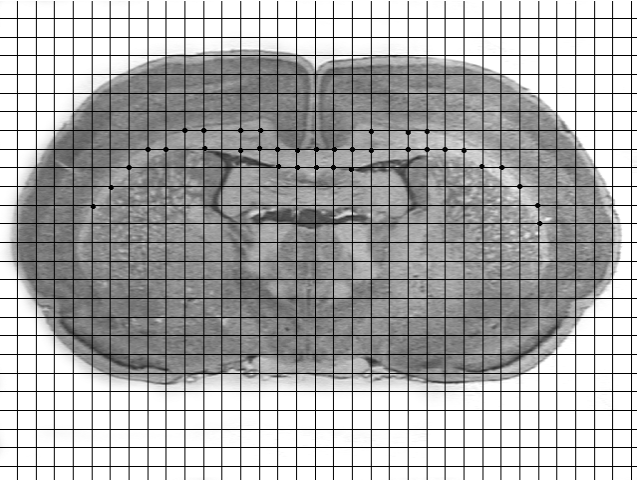
Grid overlay of a coronal section from a sham subject illustrating the point counting procedure. Each grid box is .1 mm^2^.

Between P90-93, subjects were weighed, anesthetized with ketamine/xylazine (100/15 mg/kg), and transcardially perfused with saline followed by 10% phosphate buffered formalin. Heads were removed, bottled in formalin and shipped to GDR at Beth Israel Deaconess Medical Center for histological preparation. The brains were weighed, lesions visually assessed and lesion location was confirmed. Celloidin embedding was used on whole brain tissue and sections were made in the coronal plane at 30 μm. Every fifth section was mounted, stained with cresyl violet, and coversliped before shipment back to the University of Connecticut for further analysis. The volumes of corpus callosum for each age of injury group (P1, 3 & 5) and shams were visualized using a Fisher Micromaster II digital microscope with a 1.3× lens. A series of six sections were assessed per subject, starting from approximately 2.2 mm anterior to bregma and ending approximately -7 mm posterior to bregma [23]. Measurements were derived from point counting using a .1 mm^2 ^grid overlay in ImageJ (Figure 3). Point counting was conducted blindly, without the knowledge of treatment or age condition. Volumetric analysis was computed using Cavalieri's estimator of volume [24,25].

## Authors' contributions

SWT participated in the design of the study, performed the analysis and drafted the manuscript. GDR performed the freezing lesions and histological preparation of tissue and participated in the drafting of the manuscript. RHF supervised the study and assisted in the design, as well as, drafting of the manuscript. All of the authors have reviewed and approved the final manuscript.
